# Comparison of Different EHG Feature Selection Methods for the Detection of Preterm Labor

**DOI:** 10.1155/2013/485684

**Published:** 2013-12-23

**Authors:** D. Alamedine, M. Khalil, C. Marque

**Affiliations:** ^1^CNRS UMR 7338, Biomécanique et Bio-Ingénierie, Université de Technologie de Compiègne, 60200 Compiègne, France; ^2^Azm Platform for Research in Biotechnology and Its Applications, LASTRE Laboratory, Lebanese University, Tripoli, Lebanon

## Abstract

Numerous types of linear and nonlinear features have been extracted from the electrohysterogram (EHG) in order to classify labor and pregnancy contractions. As a result, the number of available features is now very large. The goal of this study is to reduce the number of features by selecting only the relevant ones which are useful for solving the classification problem. This paper presents three methods for feature subset selection that can be applied to choose the best subsets for classifying labor and pregnancy contractions: an algorithm using the Jeffrey divergence (JD) distance, a sequential forward selection (SFS) algorithm, and a binary particle swarm optimization (BPSO) algorithm. The two last methods are based on a classifier and were tested with three types of classifiers. These methods have allowed us to identify common features which are relevant for contraction classification.

## 1. Introduction

Preterm birth, that is, birth before the 37th week of pregnancy, remains a major problem in obstetrics. Children born before term present a high risk of mortality as well as health and development problems [1]. According to the World Health Organization (WHO), preterm birth rates range between 5% and 12% of births and perinatal mortality occurs in 3% to 47% of these cases in even the most developed parts of the world [2].

Delivery occurs after the onset of regular and effective uterine contractions, which cause dilation of the cervix and expulsion of the fetus. A contraction of the uterine muscle occurs due to the generation of electrical activity in a given uterine cell that spreads to other, neighboring cells. The evolution of uterine contractions, from weak and ineffective during pregnancy to strong and effective during labor, is therefore related to an increase in cellular excitability to an increase in the synchronization of the entire uterus [3].

A primary aim of pregnancy is to maintain the well-being of both mother and fetus and to keep the latter in utero as long as needed for a healthy birth. During pregnancy, the monitoring of uterine contractility is crucial in order to differentiate normal contractions, which are ineffective, from those effective contractions which might cause early dilation of the cervix and induce preterm birth. Despite increased knowledge and understanding of the phenomena involved in the onset of preterm labor, the methods currently used in obstetrics are not precise enough for an early detection of preterm birth threats. We need a more reliable method for early detection and prevention of preterm birth threats.

One of the most promising methods for monitoring uterine activity began in the 1950s and was developed in the 1980s. It is based on the study of the electrical activity of the uterus as recorded on the mother's abdominal surface [5]. The electrohysterogram (EHG) consists of the summation of the electrical activity generated by the active uterine muscle cells, plus the noise related to corrupting electrical and mechanical activities. EHG, recorded externally, has been demonstrated to be representative of the uterine electrical activity as recorded internally [6].

Many teams have extracted features from the EHG signals in order to find specific information leading to the detection of preterm birth. Firstly, linear methods in both time and frequency domains were used to extract features from the EHG. In order to improve the results obtained by using linear methods and because the EHG, like other biomedical signals, presents some nonlinear characteristics, several measures have been proposed for detecting nonlinear characteristics in the EHG.

A large number of features have thus far been extracted from the EHG signal by many different researchers using very different population and recording protocols. In general, the complexity of calculations required for diagnostic purposes increases with the number of features in play. The reduction of feature dimensionality through the elimination of irrelevant and noisy features is very important in pattern recognition. The objective of this study is to select the most significant subset, among features extracted from the bibliography, in order to discriminate pregnancy and labor contractions, with these features being computed from the same given population. In this study, we selected from the bibliographic data 20 features (16 linear and 4 nonlinear) extracted from the EHG: mean frequency (MPF) [7], peak frequency (PF) [8–10], and deciles (*D*1 … *D*9) [11] which contain the median frequency [9, 11, 12], parameters extracted from wavelet decomposition (*W*1 … *W*5) [13], Lyapunov exponent (LE) [14, 15], time reversibility (Tr) [15], sample entropy (SE) [12], and variance entropy (VarEn) [16].

In this work, three methods are presented for feature subset selection. The first one, developed in this work, is based on the measurement of the Jeffrey divergence (JD) distance between the parameter histograms computed from the pregnancy and labor EHG classes [17]. The last two methods, developed for data mining, rely on the combination of a classifier and a search procedure: either sequential forward selection (SFS) [18] or binary particle swarm optimization (BPSO) [19]. The goal of these methods is to select, from a given feature set, the features subset that gives the maximum classification accuracy.

This paper is organized as follows. In the first part we will describe the experimental protocol. Then we will present the features extracted from the EHG processing bibliography and the three methods for feature selection. Finally, we will present the results of feature selection.

## 2. Experimental Protocol

In our study we used signals recorded on 48 women: 32 during pregnancy (33–39 weeks of gestation) and 16 during labor (39–42 weeks of gestation). The measurements were performed at two hospitals in France and Iceland. In Iceland, the measurements were performed at the Landspitali University Hospital, using a protocol approved by the relevant ethical committee (VSN02-0006-V2). In France, the measurements were performed at the Center for Obstetrics and Gynecology (Amiens), using a protocol approved by the relevant ethical committee (ID-RCB 2011-A00500-41)). After the recording, we followed the pregnant women in order to label the signals as either pregnancy or labor. When the woman gave birth within 24 hours, the signal was labeled “labor”. If the delivery occurred later, the signal was labeled “pregnancy”. In our study not all pregnancies ended by a spontaneous delivery, and in both hospitals different drugs are routinely used for labor induction or progress. In our study, 7 women received oxytocin (79 contractions), 3 women received Prostaglandin (49 contractions), 1 woman received Epidural (3 contractions), and 8 women received no drugs (38 contractions). Our database contains only singleton pregnancies.

**Figure 1:**
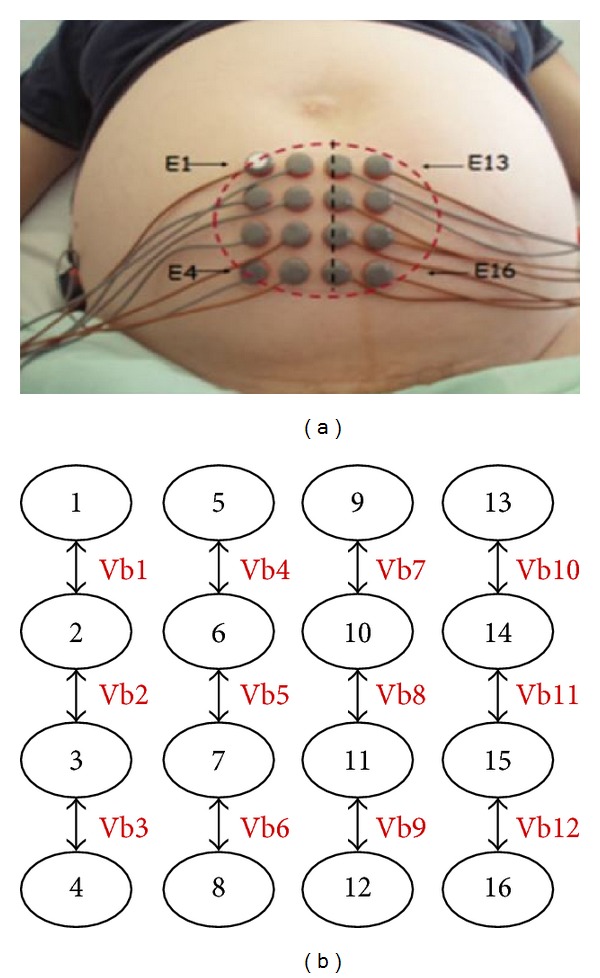
(a) Position of the 16 monopolar electrodes [4]. (b) Vb*i* (*i* = 1–12) represent the 12 calculated bipolar signals.

The EHGs were recorded using a multielectrode system composed of 18 electrodes: 16 arranged in a 4 × 4 matrix positioned on the woman's abdomen and two reference electrodes placed on each of her hips [20]. The amplifier bandwidth is 0.16–128 Hz. To increase the signal-to-noise ratio, we calculated the vertical bipolar signals (Vb*i*). Finally, we obtained 12 bipolar signals as shown in Figure 1. The bandwidth of our signal lies between 0.1 and 3 Hz. The sampling frequency used is 200 Hz, downsampled by a factor of 12 to obtain a new signal of 16.67 Hz.

In this study, we used only one bipolar signal, Vb7, because this signal is a reference recording position that has been used for a long time in our research. It is located on the median vertical axis of the uterus. The signal energy in this area remains high throughout the pregnancy as well as during labor. The bursts of uterine electrical activity that correspond to contractions were manually segmented, based on the tocodynamometer signal recorded simultaneously. After this manual segmentation of EHG bursts, we obtained a database containing 133 pregnancy bursts and 133 labor bursts.

## 3. Materials and Methods

### 3.1. Parameters Extraction

In our study 20 parameters have been extracted from the EHG. These parameters are divided into two categories: linear and nonlinear.

#### 3.1.1. Linear Parameters****

*Parameters Related to the Power Spectral Density*. Several frequency parameters have been extracted from the power spectral density (PSD), *S*_*x*_(*f*). In our work, we use the Welch Periodogram method to calculate the power spectral density of each burst [11]. This Welch Periodogram uses a window of type nfft, with size equal to the length of signal/2, with 50% overlap, for a total of three windows used. Eleven frequency parameters are extracted from this PSD: mean frequency MPF [7], peak frequency (PF) [8–10], and deciles *D*1 … *D*9 [11], which contain the median frequency *D*5 [9, 11, 12]. Deciles correspond to the frequencies *D*1 … *D*9 that divide the power spectral density into parts with each containing 10% of the total energy. Consider the following:

(1) $$\begin{array}{c} \int_{D_{P-1}}^{D_{P}}S_{x\;}\left(f\right)df=0.1\int_{0}^{f_{\max}}S_{x\;}\left(f\right)df. \end{array}$$

**Figure 2:**
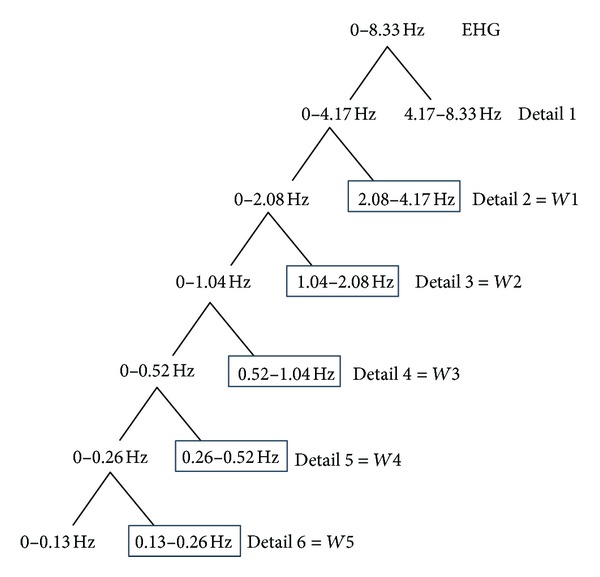
Wavelet decomposition.

*Parameters Extracted from Wavelet Decomposition. *Some authors have also used time-frequency methods, such as wavelet decomposition, to characterize the nonstationary characteristics of the EHG. In our work, we used the wavelet symlet 5, a choice based on the study referenced in [21]. This study compared several types of wavelets. The results have shown that the symlet 5 appears to be the most appropriate wavelet for the analysis of EHG signals for detection and classification purposes. After decomposition of each EHG burst into detail coefficients, we calculate the variances on the following detail levels: 2, 3, 4, 5, and 6 (named *W*1, *W*2, *W*3, *W*4, and *W*5) as previously proposed in [13]. These detail coefficients are as follows: *D*2 [2.08–4.17 Hz], *D*3 [1.04–2.08 Hz], *D*4 [0.52–1.04 Hz], *D*5 [0.26–0.52 Hz], and *D*6 [0.13–0.26 Hz] (see Figure 2). The choice of the details depends on the sampling frequency of the signal (sample rate equal to 16.67 Hz after downsampling) in order to correspond to the same frequency bands as the one selected in [13]. These selected details represent more than 96% of the signal energy and cover the frequency band of interest.

#### 3.1.2. Nonlinear Parameters****

*Time Reversibility (Tr). *A time series is reversible if the probabilistic properties are unchanged with respect to time reversal. Time irreversibility is a good indicator of nonlinearity. To calculate the time reversibility (Tr) of the signal *x* we have used equation described in [15] as follows:

(2) $$\begin{array}{c} \mathrm{Tr}\left(\tau \right)=\left(\frac{1}{N-\tau }\right)\sum_{n=\tau +1}^{N}{\left(x_{n}-x_{n-\tau }\right)}^{3}, \end{array}$$

where *N* is the signal length and *τ* is the time delay.

*Lyapunov Exponent. *The Lyapunov exponent (LE) studies the stability and the sensitivity to initial conditions of the system. It measures the rate of trajectory separation between adjacent tracks in the phase space [14, 15]. In our study we used the equation of LE described in [15] and represented by

(3) $$\begin{array}{c} \lambda =\underset{t\to \infty }{\lim}\underset{\;||\Delta_{d_{0}}||\to 0}{\lim}\left(\frac{1}{t}\right)\log\left(\frac{||\Delta_{d_{t}}||}{||\Delta_{d_{0}}||}\right), \end{array}$$

where ||Δ_*d*_0__|| represents the Euclidean distance between two states of the system at an arbitrary time *t*_0_ and ||Δ_*d*_*t*__|| corresponds to the Euclidean distance between the two states of the system at a later time *t*. 

*Sample Entropy. *We used the sample entropy (SE) to identify the regularity of EHG signals. In our work, we used the sample entropy described in [12]. A less predictable time series presents higher sample entropy. Consider a time series *x*(*t*) of length *N* and patterns *a*_*j*_(0, …, *m* − 1) of length *m*, with *m* < *N*, and *a*_*j*_(*i*) = *x*(*i* + *j*); *i* = 0, …, *m* − 1; *j* = 0, …, *N* − *m*. The time series *x*(*t*) in a time *t* = *ts*, *x*(*ts*, …, *ts* + *m* − 1) is a match for a given pattern *a*_*j*_, if |*x*(*ts* + *i*) − *aj*(*i*)| ≤ *r*, for each 0 ≤ *i* < *m*. Sample entropy is then computed as follows:

(4) $$\begin{array}{c} {\text{SE}}_{m,r\;}\left(x\right)=\{\begin{array}{cc} -\log\left(\frac{C_{m}}{C_{\left(m-1\right)}}\right), & C_{m}\neq 0\wedge C_{m-1}\neq 0,\\ -\log\left(\frac{\left(N-m\right)}{N-m-1}\right), & C_{m}=0\vee C_{m-1}=0, \end{array} \end{array}$$

where the four parameters *N*, *m*, *r*, and *C*_*m*_ represent, respectively, the length of the time series, the length of the sequences to be compared, the tolerance for accepting matches, and the number of pattern matches (within a margin for *r*) that is constructed for each *m*.

In our study, the value of *m* equals 2. This value is determined by the method of the false nearest neighbors (FNN); the value of *r* equals 0.2 according to the literature [12].

*Variance Entropy. *Recent studies have used the variance entropy (VarEn) to study biological signals but not for the EHG. We are interested in using variance entropy because this method combines the variance with sample entropy via inverse-variance weighting. For a time series *x*, variance entropy is defined as

(5) $$\begin{array}{c} \text{VarEn}\left(x,m,r\right)=\frac{\sum_{i=1}^{p}{\text{SE}}_{m,r\;}\left(x_{i}\right)\times w_{i}}{\sum_{i=1}^{p}w_{i}}, \end{array}$$

where *x*_*i*_ is the *i*th segment of *x*, *w*_*i*_ is the inverse variance of *x*_*i*_, and *p* is the number of sliding windows. *p* is not fixed because the length of signals in our database depends on EHG burst durations.

The sliding window, of size equal to 50 (window size), slides over time with a step of 45 (step size), leading to an overlap between the sliding windows which is equal to 5. The choices of window size and step size were made empirically after several trials. *p* therefore depends on window size.

Because variance entropy combines the variance with sample entropy via inverse-variance weighting, the number of windows is very important and can significantly affect the results. *p* must be neither too high nor too small. A too large *p* value induces large computing time and does not give a precise result. A too small *p* value limits detection of variability.

### 3.2. Feature Selection Techniques

#### 3.2.1. Feature Selection Based on Jeffrey Divergence Distance

This method consists of calculating, for each feature, the Jeffrey divergence (JD) distance between the two histograms obtained from the pregnancy and labor burst classes. This distance between the two histograms allows us to measure the similarity/dissimilarity of their corresponding statistical properties. A smaller distance means a larger similarity while a larger distance implies a lower similarity [22]. The divergence distance is then used to select the discriminating features. Indeed, the greater the distance between the feature histograms of pregnancy and labor classes is, the more discriminating the feature is [17].

This method has two parts. The first part consists of calculating parameters and their histograms. The second part consists of computing the distance between the histograms.

*Calculating Parameters and Their Histograms*. For each contraction of each group, we apply the following steps.

1. Calculate the nonlinear parameters on the whole EHG. These methods are time reversibility (Tr), Lyapunov exponent (LE), sample entropy (SE), and variance entropy (VarEn).
2. Calculate the variances on the following details levels after wavelet decomposition 2, 3, 4, 5, and 6 (*W*1, *W*2, *W*3, *W*4, and *W*5).
3. For each signal, compute the frequency parameters: deciles (*D*1, *D*2, *D*3, *D*4, *D*5, *D*6, *D*7, *D*8, and *D*9), mean frequency (MPF), and peak frequency (PF), from the PSD of EHG.
4. Group these values in a matrix calculated on the whole EHG database. Thus, we obtain, for a given contraction, a vector with dimension 20. The 20 columns of the matrix correspond to the 20 parameter values computed from one EHG. As we have 133 contractions in each class, we obtain 133 vectors of dimension 20 by class (one 133 × 20 matrix for each class). We then compute from these 133 vectors of each class the histogram for each parameter, giving us 2 sets of 20 histograms, one for each parameter and for each class.

*Distance between Histograms*. After obtaining, for a given parameter, the two histograms for the labor and pregnancy classes, we measure the distance between the histogram of the two classes. To measure this distance, we use the Jeffrey divergence method presented in [22]:

(6) $$\begin{array}{c} D_{Je}\left(H,G\right)=\sum_{y}\left(h_{y}\log\frac{h_{y}}{g_{y}}\;+g_{y}\log\frac{g_{y}}{h_{y}}\right), \end{array}$$

where *H* and *G* are the two histograms and where *N* bins (*N* = 10) are defined as *H* = {*h*_*y*_} and *G* = {*g*_*y*_}, with the bin index *y* ∈ {1,2,…, *N*}. After calculating the distances between every two corresponding parameter histograms for the 20 parameters, we obtain a distance vector of dimension 20. We compute the distribution of the distances contained in this distance vector. The goal of our study is to select the most discriminating parameters; therefore, we apply a threshold on the vector of distances in order to select the parameters associated with the larger distances. After verification of the Gaussianity of this distribution by using the Lilliefors test, the threshold is chosen to be equal to mean +1* standard deviation of the distance distribution.

#### 3.2.2. Sequential Forward Selection (SFS)

Sequential forward selection (SFS) is a sequential search algorithm for feature selection [23] developed for data mining. SFS begins with an empty subset. The value of the criterion function (*J*) is calculated for each feature by using a classifier. The feature presenting the best classification performance is selected (*Y*_*k*_) and then added to the subset. The next step consists of adding sequentially the feature *x* ^+^ that has the highest criterion function *J*(*Y*_*k*_ + *x* ^+^) when combined with the features *Y*_*k*_ that have already been selected. This cycle is repeated until no criterion improvement is obtained when extending the current subset. The following steps present the algorithm of SFS [18].

1. Start with an empty subset *Y*_0_ = {Φ}
2. Select the next best feature: *x* ^+^ = arg max⁡[*J*(*Y*_*k*_ + *x* ^+^)]
3. If *J*(*Y*_*k*_ + *x* ^+^) > *J*(*Y*_*k*_)
  - Update *Y*_*k*+1_ = *Y*_*k*_ + *x* ^+^, *k* = *k* + 1
  - Go to step 2
4. End

For our study, three classical classifiers have been used to compute the criterion function *J* (minimal error). The classifiers are as follows: linear discriminant analysis (LDA) [24], quadratic discriminant analysis (QDA) [25], and *K*-nearest neighbors (KNN) with *K* = 11 (the choice of *K* is based on the number of training sets) [24]. The SFS algorithm searches sequentially for the best feature subset. We then chose only the combination of features that presents this minimal error.

The SFS algorithm is applied to synthetic data to test its efficiency. The synthetic data consists of a matrix 400∗6 (400 observations corresponding to two classes defined by 6 features). Four features (features 1, 2, 4, and 6) are generated randomly (centered normalized Gaussian). The remaining ones (features 3 and 5) are generated using normalized Gaussian distributions of mean m1 for class 1 and m2 for class 2. After verification of its efficiency on synthetic data, we have applied it to our EHG database. We used 70% of the data set for classifier training and the remaining 30% for testing.

#### 3.2.3. Binary Particle Swarm Optimization (BPSO)

Particle swarm optimization (PSO) was developed by Eberhart and Kennedy in 1995. It is a population-based stochastic optimization technique that was inspired by the social behavior of bird flocking or fish schooling [26]. PSO uses a number of particles (the swarm) moving around in the search space in order to achieve the best solution. We assume that our search space is *n*-dimensional and that each particle is a point in this space. The position of the *i*th particle of the swarm is represented as *X*_*i*_ = (*x*_*i*1_,…*x*_*id*_,…*x*_*in*_). Each particle has a best previous position *p*best*i* = (*p*_*i*1_,…, *p*_*id*_,…*p*_*i*,*n*_),which corresponds to the best fitness value (in our case best classification given by a classifier fed with the selected features). The global best particle among all the particles in the population is represented by *g*best = (*p*_*g*1_,…, *p*_*gd*_,…, *p*_*gn*_). The velocity of the *i*th particle is denoted by *V*_*i*_ = (*v*_*i*1_,…*v*_*id*_,…, *v*_*in*_). The particles velocity and position are manipulated according to the following two equations:

(7) vidk+1=wvidk+c1r1k(pidk−xidk) +c2r2k(pgdk−xidk),

(8) $$\begin{array}{c} x_{id}^{k+1}=x_{id}^{k}+v_{id}^{k+1}, \end{array}$$

where *w* is the inertia weight, *c*_1_ and *c*_2_ are positive constants, and *r*_1_ and *r*_2_ are two random values in the range [0, 1].

Kennedy and Eberhart also proposed a binary particle swarm optimization (BPSO) in order to solve optimization problems with discrete valued parameters [19]. In BPSO, the position of each particle is represented as binary strings. By comparing PSO and BPSO we found that they have a common velocity equation and a different particle position equation which can be computed as follows:

(9) $$\begin{array}{c} S\left(v_{id}^{k+1}\right)=\frac{1}{1+e^{v_{id}^{k+1}}}, \end{array}$$

(10) $$\begin{array}{c} x_{id}^{k+1}=\{\begin{array}{cc} 1 & \text{if}\;r_{3}<S\left(v_{id}^{k+1}\right)\\ 0 & \text{otherwise}, \end{array} \end{array}$$

where *S*(*v*_*id*_^*k*+1^) is the sigmoid function and *r*_3_ is a random number in the range [0, 1].

BPSO has been widely used recently in the literature for feature subset selection [27]. In this case, the length of a binary string of each particle is equal to the length of the total number of features, and each particle presents a candidate for subset selection. If the bit included in the binary strings has a value of “1”, the feature is selected; otherwise, the feature is not selected. The following steps present the BPSO algorithm.

1. Initialize all particles positions and velocities randomly. Set the number of iterations *K* and other BPSO parameters.
2. Calculate the fitness value *F*(*X*_*i*_) of each particle. Fitness represents the percentages of correct classification.
3. Compare the fitness of each particle to its best fitness so far (*p*best_*i*_^*k*^of last iteration *k*):
   if  *F*(*X*_*i*_^*k*+1^) > *F*(*p*best_*i*_^*k*^)  then  *F*(*p*best_*i*_^*k*+1^) = *F*(*X*_*i*_^*k*+1^)  and  *p*best_*i*_^*k*+1^ = *X*_*i*_^*k*+1^
   Else  *F*(*p*best_*i*_^*k*+1^) = *F*(*p*best_*i*_^*k*^)  and  *p*best_*i*_^*k*+1^ = *p*best_*i*_^*k*^
4. Determine the global best position *g*best^*k*+1^ from all *p*best_*i*_^*k*+1^. Then compare *g*best^*k*+1^ with *g*best^*k*^:
   if  *F*(*g*best^*k*+1^) > *F*(*g*best^*k*^)  then  *g*best = *g*best^*k*+1^
   Else  *g*best = *g*best^*k*^
5. Update the position and the velocity of each particle according to (7) and (10).
6. Go to step 2, and repeat until the number of iterations is reached.

When the limit number of iterations is reached, we obtain an optimal solution (best subset of feature selection).

The parameters for the BPOS were chosen classically as 30 particles, with the length of each particle being equal to 20 (maximum number of features), *K* = 100 iterations. The acceleration constants *c*_1_ and *c*_2_ were set to 2. We also used a linear descending inertia weight passing from 0.6 to 0.1.

In our paper, for BPSO algorithm, three classical classifiers have been used to compute the fitness: linear discriminant analysis (LDA) [24], quadratic discriminant analysis (QDA) [25], and *K*-nearest neighbors (KNN) with *K* = 11 (the choice of *K* is based on the number of training sets) [24]. The best feature subset chosen by the BPSO algorithm is defined as the one giving the maximum percentages of correct classification after 100 iterations (1 run). Then, to evaluate the performances and variability of BPSO, we performed multiple runs (200 runs). This algorithm is applied to the same synthetic and real data as described above.

## 4. Results

### 4.1. Results of Parameters Extraction

For each contraction of each group, we calculate the 20 parameters (linear and nonlinear parameters) from the EHG.  Table 1 presents the mean ± standard deviation of each of the parameters in each class. Additionally, in this table we present for each parameter the Lilliefors test result concerning its Gaussianity.

### 4.2. Results of Feature Selection Using JD Distance

**Figure 3:**
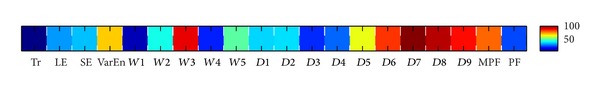
Color vector representing the distribution of distances between parameters.

We first present the results obtained with the feature selection method based on the Jeffrey divergence. After calculating the distances between every two corresponding feature histograms for the 20 features, we obtain a distance vector of dimension 20, as presented in Figure 3. The red color represents the maximum distance value of the distribution and the blue color its minimum. Each coordinate of this vector represents a different feature.

**Figure 4:**
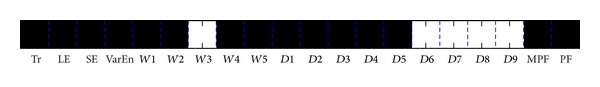
Selection vector representing the best parameters for the discrimination between pregnancy and labor.

Figure 4 shows the selection vector obtained after applying the threshold (equal to the mean +1* standard deviation of the distance distribution) on the distance vector. A white color indicates that the feature has been selected as being discriminating between pregnancy and labor.

With this approach we selected 5 discriminating features: variance on the wavelet decomposition detail level 4 (*W*3), decile 6 (*D*6), decile 7 (*D*7), decile 8 (*D*8), and decile 9 (*D*9).

### 4.3. Results of Feature Selection Using SFS and BPSO

#### 4.3.1. Results on Synthetic Data

The algorithms BPSO and SFS were first applied to the synthetic data described above in order to test their efficiency.  Table 2 presents the results obtained after applying SFS and BPSO to these synthetic data. We notice that the two features 3 and 5 marked in bold font in Table 2 are always selected by the two algorithms (BPSO and SFS) whatever the mean m1 and m2 (m1#m2) and whatever the classifier. We can also notice that both methods select larger sets than the minimum set containing the 2 clearly discriminating features, whatever the classifier, with SFS giving smaller sets than BPSO most of the time.

#### 4.3.2. Results on Real Uterine EMG Signals

Table 3 presents the selected feature subset obtained from BPSO and SFS when applied to our EHG database by using the 3 different classifiers. Each selected feature subset corresponds to that giving the maximum percentage of correct classification (93.73% with QDA, 91.23% with LDA, and 89.97% with KNN). Table 3 also presents the best feature subsets as selected by SFS, which corresponds to the minimal error.

From Table 3, we can notice that BPSO always selects 3 features regardless of classifier type. These features are VarEn, D1, and D8. For SFS, the common features between the subsets obtained from the three classifiers are SE, VarEn, W2, and D8. Only VarEn and D8 marked in bold font in Table 3 are systematically selected by both methods.

### 4.4. Validation

The results presented above give seven subsets of features selected by using JD, SFS with QDA, SFS with LDA, SFS with KNN, BPSO with QDA, BPSO with LDA, and BPSO with KNN. We then evaluated the performances of these seven selected subsets by calculating the percentages of correct classification that they give when used as inputs of a classifier. We used for this validation the same classifiers as used for the selection phase: QDA, LDA, and KNN.

Table 4 presents the percentages of correct classification for each subset by using the QDA classifier. The subset of features selected by BPSO with QDA presents the highest percentage of classification (88.72%) followed by the one selected by SFS with QDA (87.47%).  Table 5 presents the percentage of correct classification for each subset by using the LDA classifier. The result indicates that the subset selected by SFS with QDA and SFS with KNN presents the highest percentage (84.96%) followed by the ones selected by SFS with LDA and BPSO with LDA (83.71%).  Table 6 presents the percentage of correct classification for each subset by using the KNN classifier. The result indicates that the subset selected by BPSO with QDA presents the highest percentage of classification (87.47%) followed by the ones selected by BPSO with KNN (84.96%).

## 5. Discussions and Conclusions

In this paper, we have extracted several features (linear and nonlinear) from the EHG. Then we have applied three selection techniques (Jeffrey divergence distance, SFS, and BPSO) in order to select the most pertinent features allowing discrimination between labor and pregnancy contractions.

It is clear from Table 2 (results obtained for synthetic data) that the algorithms BPSO and SFS have the ability to select discriminating features. In Table 3, which presents the selection results obtained from EHG signals, we notice that BPSO and SFS selected different subsets of features when using the three different classifiers. It is very important to highlight the most repetitive features selected with the different methods. Indeed, these features are expected to be very pertinent. Five features have been selected when applying the JD algorithm (W3, D6, D7, D8, and D9). Three others have been repeatedly selected by BPSO (VarEn, D1, and D8) and three others have been repeatedly selected by SFS (SE, VarEn, W2, and D8), whatever the type of classifier. Only D8 is common to these 3 subsets. D8 corresponds to the decile of mean value 0.36 ± 0.09 for the pregnancy class and 0.45 ± 0.12 for the labor class (Table 1). This increase in D8 value is in agreement with the most accepted observation made by teams that have worked on EHG frequency content, whatever the species: a clear shift towards higher frequencies of the EHG frequency content when going from pregnancy to labor [3, 6].

Comparing the 6 subsets of features obtained by BPSO and SFS, we notice that two features, VarEn and D8, are also common. This confirms the observation made by different teams concerning the interest of taking into account the nonlinear characteristics of EHG for diagnostic purposes [12, 14, 15]. VarEn also increases from pregnancy to labor (Table 1), indicating an increase in EHG nonlinearity from pregnancy to labor, which is in agreement with the work done by different teams [12, 14, 15]. VarEn performed better here than the other nonlinear features computed by these teams. This short subset should also be of diagnostic interest.

From the validation study, developed in order to test the performance of the selected subsets, we notice from Table 4 that the feature selected by BPSO with QDA corresponds to the highest percentage of correct classification (88.72%) obtained when using QDA. Table 6 presents the second highest percentage of correct classification (87.47%) obtained with the subset of features selected by BPSO with QDA by using KNN. This allows us to say that BPSO associated with QDA seems to be the most efficient feature selection method in our study.

Two conclusions can be drawn from these results.

- The most discriminating subset should be (or at least should contain) the 2 following features: VarEn and D8. Indeed, they are the most pertinent features selected in the 7 subsets of features defined by using JD, SFS, and BPSO, whatever the classifier.
- BPSO with QDA gives larger selected sets than the JD method, which might be demanding in time and in training data. But these sets also give much better results for the validation phase when using nonlinear classifiers. This tends to imply that the classification of EHG should be based on a nonlinear approach, either for feature selection or classification, rather than on linear ones.

As future work, we will classify the EHG by using, as inputs of the classifier, only the selected features in order to compare the obtained results with the ones obtained by using all the features. We will also test the best methods of feature selection on a larger database. Accordingly, we will be able to use more robust and relevant cross-validation techniques than the simple one used in this study. Additionally, we should try other classification methods for the validation phase, such as those based on neural networks, SVM, or KNN. We will also include in this selection process features related to uterine synchronization and activity propagation that have been proven to beof interest for EHG monitoring [4], as soon as they are available from the work currently in progress in our team. With this work we expect to obtain the most pertinent data analysis features for predicting the preterm labor threat as early as possible.

## Figures and Tables

**Table 1:** Mean ± standard deviation (STD) of parameters and results of Gaussianity test.

Parameter	Mean ± STD (pregnancy)	Gaussian	Mean ± STD (labor)	Gaussian
**Tr**	0.001 ± 0.01	N	−0.0001 ± 0.01	N
**LE**	5.47 ± 0.63	Y	5.33 ± 0.52	Y
**SE**	1.17 ± 0.19	Y	1.23 ± 0.18	Y
**VarEn**	0.61 ± 0.30	Y	0.71 ± 0.29	Y
***W1***	0.0084 ± 0.0076	N	0.0118 ± 0.0114	Y
***W2***	0.03 ± 0.02	N	0.04 ± 0.03	Y
***W3***	0.07 ± 0.04	N	0.13 ± 0.09	Y
***W4***	0.27 ± 0.11	N	0.30 ± 0.10	Y
***W5***	0.48 ± 0.11	Y	0.41 ± 0.13	Y
***D1***	0.14 ± 0.02	N	0.15 ± 0.02	Y
***D2***	0.16 ± 0.03	N	0.17 ± 0.04	N
***D3***	0.18 ± 0.03	N	0.20 ± 0.05	N
***D4***	0.20 ± 0.04	N	0.23 ± 0.07	N
***D5***	0.22 ± 0.05	N	0.27 ± 0.08	N
***D6***	0.25 ± 0.06	N	0.32 ± 0.10	Y
***D7***	0.29 ± 0.07	N	0.37 ± 0.10	Y
***D8***	0.36 ± 0.09	N	0.45 ± 0.12	Y
***D9***	0.52 ± 0.18	N	0.64 ± 0.22	Y
**MPF**	0.30 ± 0.06	N	0.35 ± 0.08	Y
**PF**	0.18 ± 0.07	N	0.21 ± 0.10	N

**Table 2:** Results of BPSO and SFS on synthetic data. The features marked in bold font, correspond to the discriminating features.

Classifiers	BPSO (gbest that have best fitness)	SFS (combination of features with minimal error)
QDA	[**3**, 4, **5**]	[**3**, **5**, 6]
LDA	[1, **3**, 4, **5**, 6]	[**3**, **5**, 6]
KNN	[**3**, 4, **5**, 6]	[2, **3**, **5**]

**Table 3:** Comparison between BPSO and SFS. The common features between the subsets obtained from BPSO and SFS by using the three classifiers are marked in bold font.

Classifier	BPSO (gbest that have best fitness between 200 runs)	SFS (combination of features with minimal error)
QDA	LE, **VarEn**, *W1, W4, D1, D2, D4, D5, * ***D8*** *, D9 *	TR, LE, SE, **VarEn**, *W1, W2, W4, * *** D8,*** * D9 *
LDA	SE, **VarEn**, *W1, W2, D1, D4, * ***D8***, MPF	SE, **VarEn**, *W2, W3, D1, D3, D7, * *** D8,*** * D9,* MPF
KNN	LE, SE, **VarEn**, *W3, W4, D1, D3, D5, D6, * ***D8***	TR, LE, SE, **VarEn**, *W1, W2, W3, W4, W5, D1, D2, D3, D4, D7, * *** D8,*** MPF

**Table 4:** Comparison of the percentage of correct classification of the selected features subset by using QDA.

Selection method	Selected feature subset	Correct classification using QDA
JD	*W3, D6, D7, D8, D9 *	79.95%
SFS with QDA	TR, LE, SE, VarEn, *W1, W2, W4, D8, D9 *	87.47%
SFS with LDA	SE, VarEn, *W2, W3, D1, D3, D7, D8, D9,* MPF	83.71%
SFS with KNN	TR, LE, SE, VarEn, *W1, W2, W3, W4, W5, D1, D2, D3, D4, D7, D8,* MPF	84.96%
BPSO with QDA	LE, VarEn, *W1, W4, D1, D2, D4, D5, D8, D9 *	**88.72%**
BPSO with LDA	SE, VarEn, *W1, W2, D1, D4, D8,* MPF	81.20%
BPSO with KNN	LE, SE, VarEn, *W3, W4, D1, D3, D5, D6, D8 *	86.22%

**Table 5:** Comparison of the percentage of correct classification of the selected features subset by using LDA.

Selection method	Selected feature subset	Correct classification using LDA
JD	*W3, D6, D7, D8, D9 *	81.20%
SFS with QDA	TR, LE, SE, VarEn, *W1, W2, W4, D8, D9 *	**84.96%**
SFS with LDA	SE, VarEn, *W2, W3, D1, D3, D7, D8, D9,* MPF	83.71%
SFS with KNN	TR, LE, SE, VarEn, *W1, W2, W3, W4, W5, D1, D2, D3, D4, D7, D8,* MPF	**84.96%**
BPSO with QDA	LE, VarEn, *W1, W4, D1, D2, D4, D5, D8, D9 *	82.46%
BPSO with LDA	SE, VarEn, *W1, W2, D1, D4, D8,* MPF	83.71%
BPSO with KNN	LE, SE, VarEn, *W3, W4, D1, D3, D5, D6, D8 *	82.46%

**Table 6:** Comparison of the percentage of correct classification of the selected features subset by using KNN.

Selection method	Selected feature subset	Correct classification using KNN
JD	*W3, D6, D7, D8, D9 *	78.70%
SFS with QDA	TR, LE, SE, VarEn, *W1, W2, W4, D8, D9 *	83.71%
SFS with LDA	SE, VarEn, *W2, W3, D1, D3, D7, D8, D9,* MPF	81.20%
SFS with KNN	TR, LE, SE, VarEn, *W1, W2, W3, W4, W5, D1, D2, D3, D4, D7, D8,* MPF	83.71%
BPSO with QDA	LE, VarEn, *W1, W4, D1, D2, D4, D5, D8, D9 *	**87.47%**
BPSO with LDA	SE, VarEn, *W1, W2, D1, D4, D8,* MPF	81.20%
BPSO with KNN	LE, SE, VarEn, *W3, W4, D1, D3, D5, D6, D8 *	84.96%
